# Editorial: Case reports in personality disorders

**DOI:** 10.3389/fpsyt.2024.1358775

**Published:** 2024-01-31

**Authors:** Massimiliano Beghi, Michele Sanza

**Affiliations:** Department of Mental Health and Addictions, Azienda Unità Sanitaria della Romagna (AUSL), Cesena, Italy

**Keywords:** personality disorders [mortality; *therapy], ECT, mood, psychotherapeutic, EMDR, treatment

Data from Waves 1 and 2 of the National Epidemiologic Survey on Alcohol and Related Conditions suggest that, in the United States, approximately 15% of the adult population suffers from at least one personality disorder (PD) (1). PDs are pervasive patterns of interior and behavioral experience that stray from cultural and individual expectations, leading to an impairment in self-functioning (identity and self-direction) and interpersonal functioning (empathy and intimacy) (2). These patterns manifest themselves in different areas like cognition, affectivity, interpersonal functioning and impulse control. PDs are characterized by the persistence of these traits over time, and in different situations. PDs are of great social impact for their high prevalence (the burden on psychiatric services is approximately 10%) (3) and high mortality, often due to suicide (4).

For these reasons, an improvement in the treatment of PDs is required to reduce their burden. So far, there is no evidence about the effectiveness of pharmacological treatment (which has only shown mild efficacy on impulse control and emotional regulation) (5), while it seems that psychological treatment is more efficacious, although there is no agreement in the literature on which treatment should be the first-line choice (6, 7). However, despite the acquisitions of recent decades that attest that personality disorders should be treated with psychotherapeutic interventions, the offering of treatment provided is predominantly anchored to pharmacotherapy alone (8).

In this Research Topic, we have presented four studies, all focused on treatment strategies for PD.

Hafkemeijer et al. presented two case reports with a diagnosis of borderline PD (BPD) and not fulfilling the diagnostic criteria for post-traumatic stress disorder (PTSD), to investigate the efficacy of trauma-focused therapy through a 10-session intensive trauma-focused therapy course using eye movement desensitization and reprocessing (EMDR) therapy, considering a bidirectional relation between the two disorders (25–30% of PTSD patients meet the diagnostic criteria for BPD, while 30–70% of BPD patients fulfilled the diagnostic criteria for PTSD in their lifetime). The authors targeted both A-criterion-worthy memories (without intrusive reliving) and non-A-criterion-worthy memories, which are considered responsible for the patients’ most prominent symptoms. Then, they investigated the effects on psychological distress, quality of life, and difficulties in emotion regulation at intake, post-treatment, and at 3, 6-and 12-month follow-up. Both patients showed a strong decline in psychological distress and difficulties in emotion regulation, and reported an improvement in their quality of life, no longer meeting the diagnostic criteria of BPD. The authors concluded that EMDR therapy appears to be a promising treatment approach for patients with BPD.

Blay et al. described an adaptation of Good Psychiatric Management (GPM), a generalist clinical management approach for borderline PD (BPD) that incorporates common ingredients of good standard care for any psychiatric diagnosis with what works from prevailing specialist psychotherapies, to allow generalist clinicians to have simple-to-use dimensional tools that may help them distinguish, in the different categorical PD diagnoses, the most relevant issues for the patient. They administered it to one case of BPD with core detachment, negative affectivity, dissociality and other three comorbid personality disorders, and another case of BPD with core anankastia in a patient also diagnosed with obsessive-compulsive PD, assuming that BPD criteria may be the strongest markers of general personality functioning. The authors hypothesized that the disorder’s symptoms arise from a dynamic model called interpersonal hypersensitivity (IHS), which provides a framework for understanding the fluctuating self- and interpersonal issue patterns prototypical of BPD. They concluded that specialist psychotherapies should therefore meet the demands of public health needs to treat personality dysfunction, and the incorporation of new dimensional models of diagnosis is needed for treatments that can provide a minimum standard of care for providers and patients.

Mu et al. described the case of a 24-year-old young woman suffering from Paranoid Personality Disorder (PPD) with clinical manifestations of feeling targeted, cheated, tracked, misunderstood, and repeating actions. Since the prescribed pharmacotherapy was not effective for her delusions, the patient underwent Modified electric convulsive treatment (MECT). After MECT, the psychopathology improved, but the patient’s body temperature increased, and leukocytosis was found. After excluding infection and other possibilities, intravenous 1000 ml of physiological saline was administered to the patient. The white blood cell (WBC) count normalized shortly. The authors concluded that MECT treatment should be considered for drug-resistant psychotic symptoms in PPD, but it is necessary to screen blood cytology.

A study by Webster et al. highlighted the validity of the Post Traumatic Model (PTM) in a female patient who suffered severe and prolonged physical, psychological, and sexual abuse from two years old through adulthood, with the development of a Dissociative Identity Disorder (DID) (four full identity “alters” in her head). Later, the patient was also diagnosed with Major Depressive Disorder (MDD), PTSD, and associated features such as passive or active suicidal ideations and psychomotor retardation, which she had not been able to cope with in almost 10 years of therapy and psychiatric medications. After a hospitalization, the patient was referred by the inpatient psychiatrist for Electroconvulsive Therapy (ECT) and after two years, she reported having “lost the others”. With the integration of these alters she was able to restore the memories and pain that the alters had protected her from. This case highlights the importance of the PTM as an etiological description for DID and the importance of mental health providers further studying and researching the effects of ECT on patients with chronic MDD, PTSD, and suicidality, especially if these are comorbid with DID.

The case reports in this Research Topic underline the scientific community’s efforts to detect efficacious treatments for PDs. They highlight the importance of collecting a detailed psychiatric history, and investigating physical and sexual trauma histories. Moreover, it is important to consider the dimensional aspects of personality instead of focusing on the categorical approach, to find the most important aspect to cope with during the psychotherapy sessions. ECT can provide some improvement in drug-resistant patients, but (even rare) side effects should be taken into consideration. Moreover it is very important to treat them through the interaction between biological and psychosocial aspects of mental illness and the complexities of this biopsychosocial model. 

## Author contributions

MB: Conceptualization, Writing – original draft, Writing – review & editing. MS: Writing – review & editing.
